# Comparative evaluation of the efficacy of customized maxillary oral appliance with mandibular advancement appliance as a treatment modality for moderate obstructive sleep apnea patients—protocol for a randomized controlled trial

**DOI:** 10.1186/s13063-022-06070-w

**Published:** 2022-02-16

**Authors:** Vikram Belkhode, Surekha Godbole, Sharayu Nimonkar, Pranali Nimonkar, Sweta Pisulkar

**Affiliations:** 1 Department of Prosthodontics, Sharad Pawar Dental College & Hospital, Datta Meghe Institute of Medical Sciences (Deemed to be University) Sawangi (Meghe), Wardha, Maharashtra India; 2 Trauma Care Centre, Government Medical College, Nagpur, Maharashtra India

**Keywords:** Apnea/hypopnea index, Continuous positive airflow, Customized maxillary oral appliance, Mandibular advancement device, Obstructive sleep apnea, Polysomnography

## Abstract

**Background:**

Obstructive sleep apnea (OSA) is due to the obstruction of the upper airway during sleep. This condition is often associated with multiple symptoms and co-morbidities. There are many treatment options mentioned in the literature to manage OSA, among which interventional option of continuous positive airflow (CPAP) and non-interventional option, i.e., mandibular advancement device (MAD), which is an oral appliance (OA), are the most preferred ones. This study aims to evaluate the efficacy of customized maxillary oral appliances with mandibular advancement devices in moderate OSA patients.

**Methods:**

A prospective interventional study with a randomized controlled trial will be carried out involving 40 participants (sample size), with an apnea-hypopnea index (AHI) > 15–30, recorded on polysomnography (PSG). Study participants will be randomly divided into the following treatment groups: control group or group subjected to mandibular advancement device (MAD, *n*=20) and second group subjected to customized maxillary oral appliance (CMOA, *n*=20). Baseline assessment of apnea/hypopnea index (AHI), oxygen saturation in blood, percentage of rapid eye movement, electroencephalogram, electrocardiogram, oro-nasal airflow via a pressure transducer, and Epworth Sleepiness Scale will be done. Then both study group participants will receive their respective appliances. And after one month and three months of delivery of the appliance, all the parameters, i.e., AHI, oxygen saturation in blood, percentage of rapid eye movement, electroencephalogram, electrocardiogram, oro-nasal airflow via a pressure transducer, and Epworth Sleepiness Scale will be re-evaluated and compared with the baseline measurements. Descriptive and analytical statistics will be done. SPSS (Statistical Package for Social Sciences) Version 20.1 will be used as statistical software. The statistical significance between the two groups after one month and three months will be evaluated at *p*< 0.05.

**Discussion:**

We expect, customized maxillary oral appliance to be more efficient in managing moderate OSA, in comparison with MAD. If the hypothesis of the present study is confirmed, then this customized maxillary appliance will be quoted as a “gold standard” for managing moderate OSA.

**Trial registration:**

CTRI/2020/07/026936 Registered 31 July 2020.

## Background

Obstructive sleep apnea (OSA) is identified by repetitive events of partial or total blockage of the upper airway during sleep resulting in arterial oxygen desaturation and arousals. This syndrome is often associated with many symptoms and co-morbidities, such as excessive daytime sleepiness, cognitive problems, obesity, type 2 diabetes mellitus, hypertension, exacerbation of the chronic obstructive pulmonary disease, apnea, nocturnal awakening, episodes of choking during sleep, and morning headache [1–3]. Severe OSA has been reported as the greatest risk factor for atherosclerosis, acute myocardial infarction, and general mortality [4].

Literature has documented OSA as an independent risk factor for cardiovascular disease, ischemic stroke, and general mortality. Surveys have proven poor quality of life among patients suffering from OSA, and also significantly higher incidences of industrial and road traffic accidents in them [5].

Polysomnography (PSG) is a diagnostic tool used for OSA. It records the apnea/hypopnea index (AHI) to determine the severity of OSA. Depending on AHI, OAS is classified as mild (AHI 5–15), moderate (AHI > 15–30), or severe (AHI > 30) [6]. Treatment options for OSA are behavioral and surgical weight loss therapies, positional therapy, pharmacological therapy, surgical therapies (Pharyngeal and maxillomandibular surgeries), Continuous positive airway pressure (CPAP), and oral appliances (OA) such as mandibular advancement device (MAD) [7–9]. Among these treatment options except for CPAP and OA, results for other non-surgical therapies are highly unsatisfactory [10]. CPAP is considered to be a gold standard treatment option for patients with OSA. At the same time, the use of CPAP is associated with various problems such as muscle sagging, discomfort to pressure sensation and its leakage, skin inflammation, the loud noise of the machine, and many more, making it noncompliant to the users [11].

MAD has become a viable alternative and the most widely accepted treatment of choice by patients suffering from mild to moderate OAS. Several authors in their studies have concluded that MAD has a good contribution in reducing AHI and in raising the quality of life in such patients when compared with CPAP [12, 13]. MAD works on the principle of clasping the mandible in a forward and downward position that helps to reduce AHI by enlarging the upper airway [14].

However many long-term model analysis studies have reported several side effects of MAD, such as tooth pain, temporomandibular joint disorders, xerostomia or excessive salivation, and gum irritation [15–17]. At the same time, some studies have shown that TMD symptoms in patients using occlusal splint last up to 3–4 months. For most patients, the symptoms disappeared after 5 years [18].

Given the high prevalence of OSA among the population and the side effects of existing appliances, new effective treatment options would be welcomed. The present study focuses on a novel OA named customized maxillary oral appliance (CMOA), to provide a new therapeutic option for patients with moderate OSA. This protocol will help to undertake the study to evaluate the efficacy of this CMOA against MAD for managing mild to moderate OSA.

## Methods

### Ethical aspects

The protocol for the present study was submitted to the institutional Ethical committee. After approval, the study was registered for a randomized controlled trial (RCT). The study participants will be informed about the study and the intervention to be carried out. Signed consent in the local language will be obtained from them before the study starts. The data obtained in the course of the trial will be treated under the applicable Data Protection Law.

A team of 4 members of the Institutional ethical committee will be appointed under Data Monitoring Committee (DMC) is to ensure the ethical conduct of the trial and to protect the safety interests of patients during and after the trial.

In situation wherein termination of the trial if required the Institutional Ethics Committee (IEC) and the Competent Regulatory Authorities will be informed.

The data obtained in the course of the trial will be treated under the applicable Data Protection Law (EU General Data Protection Regulation – GDPR - 2016/679), the Federal Data Protection Act (Bundesdatenschutzgesetz, BDSG), the State Data Protection Act of Baden-Württemberg (Landesdatenschutzgesetz, LDSG BW) as well as § 40 (2a) AMG.

During the clinical trial, patients will be identified solely utilizing an individual identification code (Patient ID). Storage of trial findings on a computer will be done by local data protection law and will be handled in the strictest confidence.

### Protocol amendments

If the trial protocol has to be changed substantially after approval, a written amendment will be required that must be signed by the same persons as mentioned in the trial protocol. Any protocol amendment will only be implemented after approval has been granted by the IEC and the competent authorities. Any substantial protocol amendment affecting the benefit-to-risk ratio must be approved by the responsible EC will be notified. Participants will be informed about relevant changes in the trial and will be asked to re-consent in writing.

### Study design (Fig. 1, Table 1)

The present study is a randomized, controlled, parallel, and two-armed (i.e., MAD and CMOA), clinical trial. The duration of the study will be for one year. The patients reporting to the Department of Sleep Medicine, which are diagnosed cases of moderate OSA will be considered as participants for this study.

**Fig. 1:**
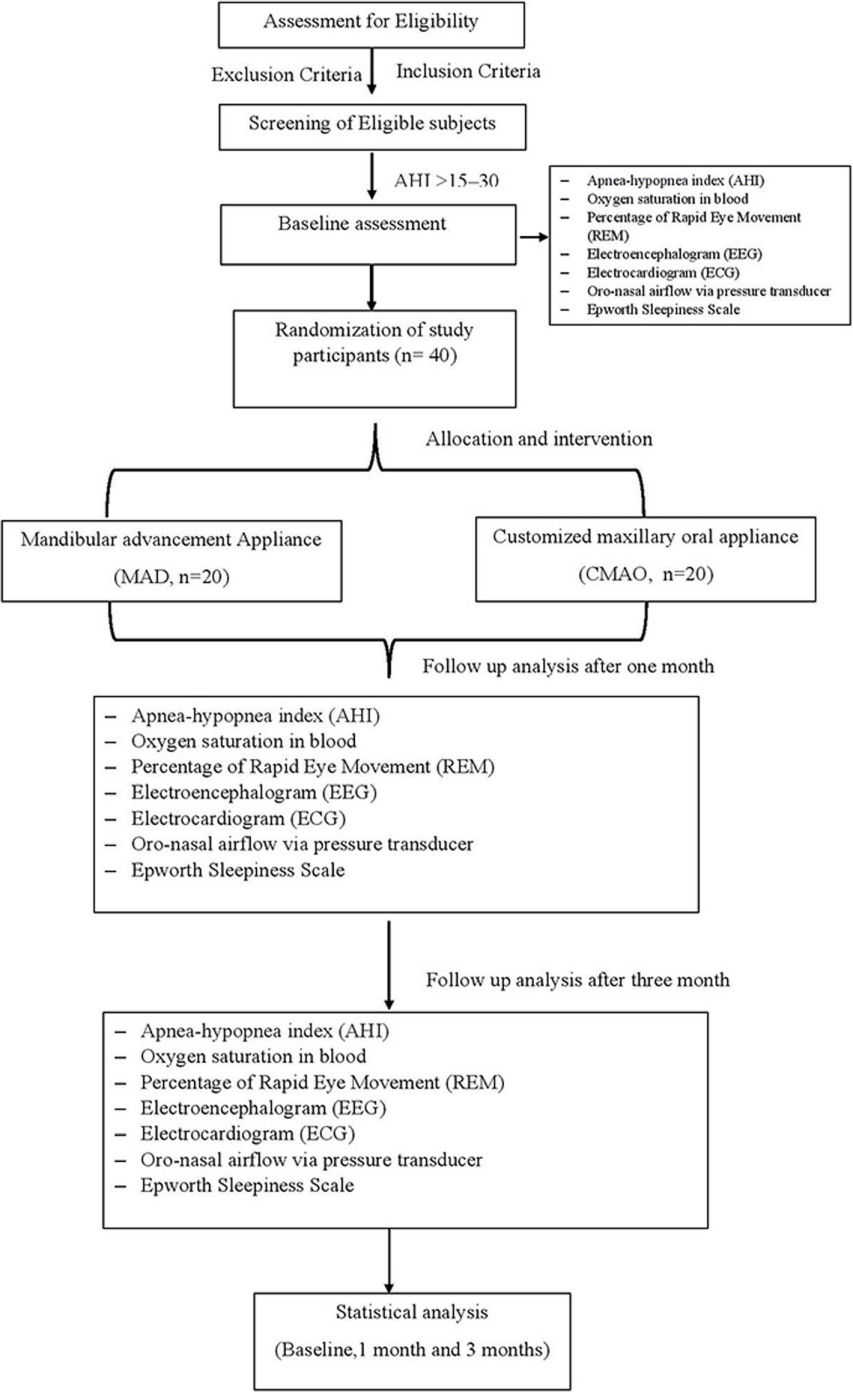
Flowchart of the study

**Table 1:** SPIRIT (Standard Protocol Items: Recommendations for Interventional Trials)

Timepoint	Pre-treatment	Treatment	Post-treatment	
			1 month	3 month
**Enrolment:**				
Eligibility screen	X			
Informed consent	X			
Screening	X			
Baseline data collection	X			
Randomize subject allocation	X			
**Interventions:**				
MAD		X		
CMOA		X		
**Assessments:**				
Primary outcome			X	
Secondary outcomes				X

The study involves 40 sample sizes. At 80% power of the study, the minimum sample size calculated for each group is 16. However, 20 samples in each group will be selected to minimize errors and to cover any loss of cases during follow-up.

These 40 participants will be randomized by creating a computer-generated randomization list. Participants will be assigned random numbers based on consecutive enrolment into group 1 or a control group subjected to MAD (*n*=20) and another group, i.e., group 2 subjected to CMOA.

### Inclusion criteria

1. The patients diagnosed with moderate OSA (apnea/hypopnea > 15–30 events/hour)
2. Patients were non-compliant to CPAP and refused surgical intervention.
3. Age ranging from 30 to 50 years
4. BMI (range 17–39 kg/m^2^)

### Exclusion criteria

1. The patient diagnosed with severe OSA
2. The patient diagnosed with mild OSA
3. Uncooperative Subjects
4. Patients with severe periodontal diseases
5. An edentulous arch, or without a sufficient number of teeth for the adequate retention of the appliance.
6. Patients with temporomandibular joint disorders
7. Patients with pathologic evidence of airway obstruction.
8. Patients with maximal protrusion of less than 6 mm

### Allocation concealment mechanism

Upon confirmation of eligibility (patients must meet all inclusion criteria and must not meet exclusion criteria), the clinical site will contact a centralized internet randomization system (https://randomizer.at/). Patients will be randomized using block randomization to one of the two arms, MAD arm or CMOA arm.

Generation of allocation sequence, enrolment of participants, and assignment of participants to interventions will be performed by authors in the Department of Sleep Medicine, DMIMS (DU).

## Intervention

### Mandibular advancement device (MAD) Fig. 2

MAD will be fabricated for the Control Group. An impression of upper and lower jaws will be made for all the participants of this group and will be poured to form a cast. A protrusion index will be recorded using a George Gauge by protruding the mandible to 50% of the maximum protrusion. (roughly 6 mm). The appliance will have an upper and lower part of the elastomer and will be interconnected with a screw that will be tightened by advancing the mandible by 6 mm from its centric position. Symptoms related to temporo-mandibular disorder (TMD) will be evaluated. All the patients will be advised to wear the appliance daily while sleeping at least for 6 h.

**Fig. 2:**
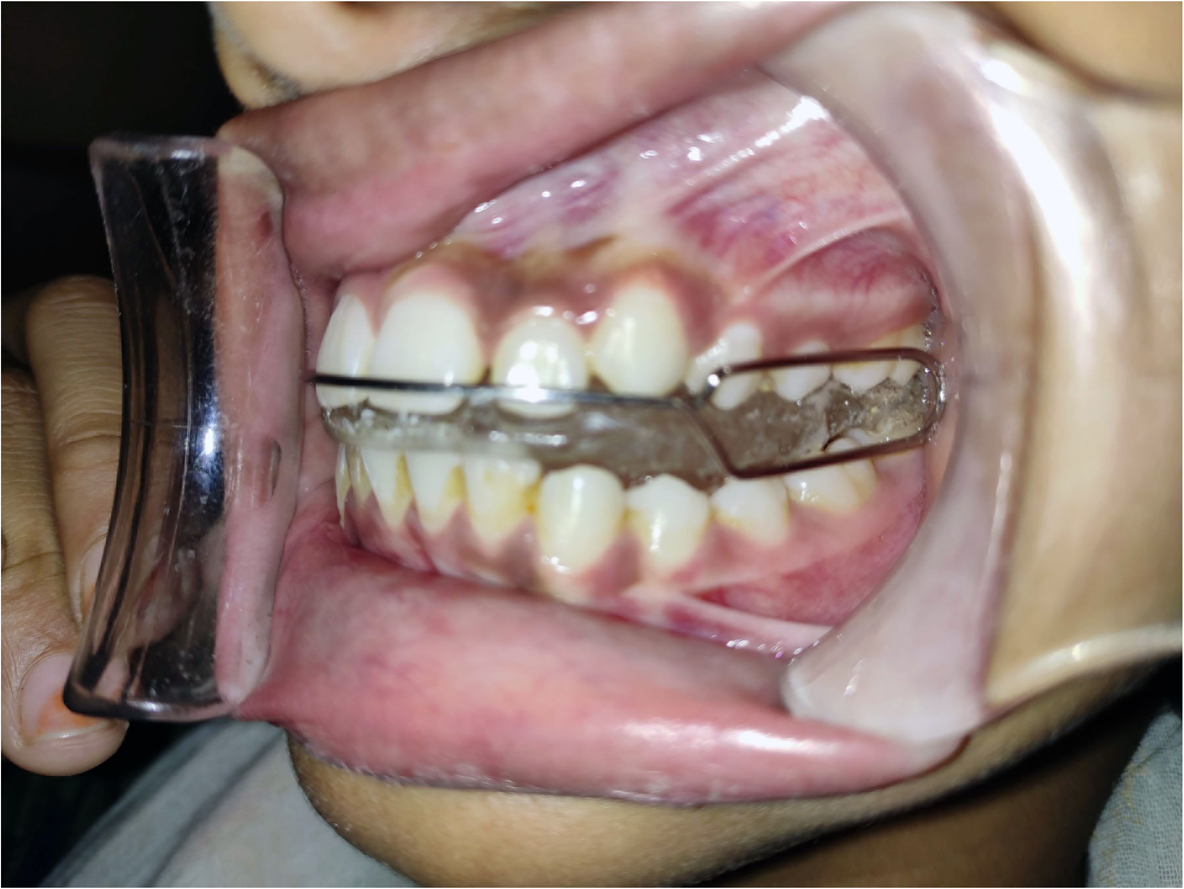
Mandibular advancement appliances

### Customized maxillary oral appliance (CMOA)

#### Design (Figs. 3, 4 and 5)

The appliances will have a base plate and an upper plate. The base plate will be adapted to the maxillary hard and soft tissues (teeth and hard palate). This plate will be overextended by 1 mm in the soft palate region to support, lift, and prevent its downfall. The upper plate of the appliance is designed over the base plate, keeping a 2 mm gap between the base plates. This gap will be made hollow. The upper plate will have the occlusal anatomy of the maxillary teeth so that it occludes with the mandibular teeth (in present occlusal relation), but at an increased vertical dimension which will be achieved by hollowing the plate. These hollow maxillary upper plates will have a hole in the anterior region, that is in the central incisor area to promote continuous inflow of the fresh air that will be directed to the posteriors region of the tongue. In addition to this, a bulge will be designed on the palatal surface of the upper plate of the appliance in the posterior region to prevent the backfall of the tongue.

**Fig. 3:**
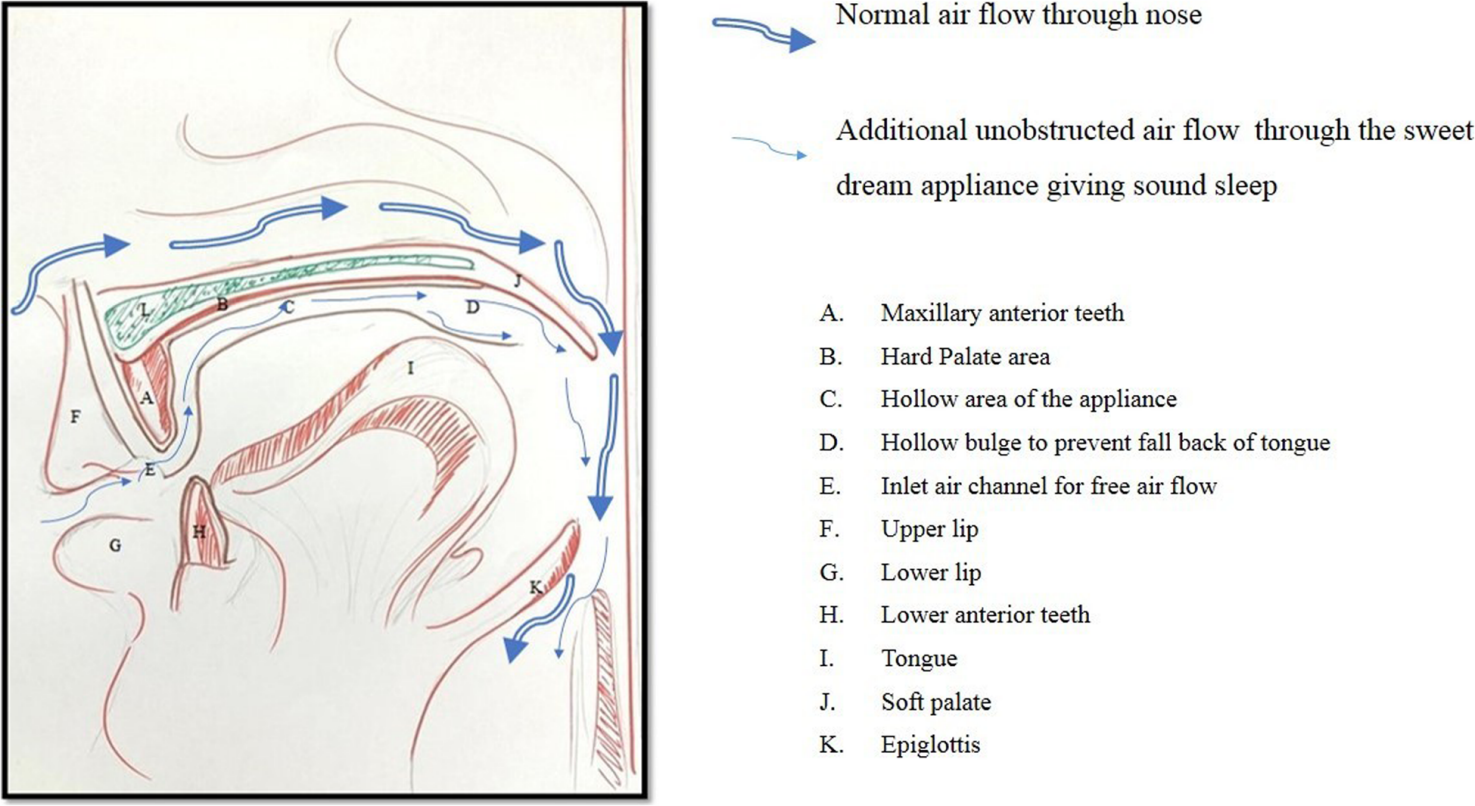
Schematic design of modified oral appliance

**Fig. 4:**
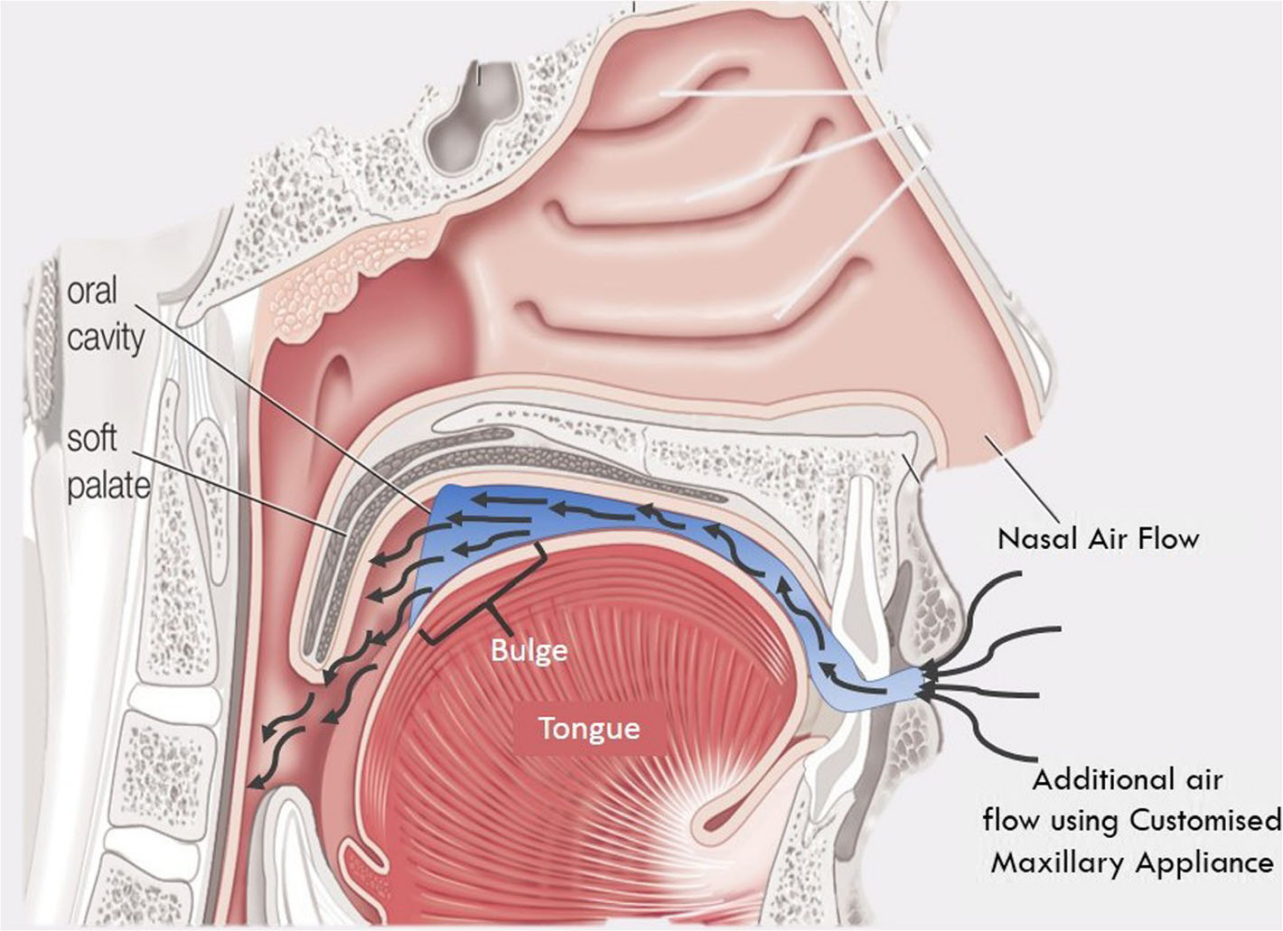
Labeled design of modified oral appliance

**Fig. 5:**
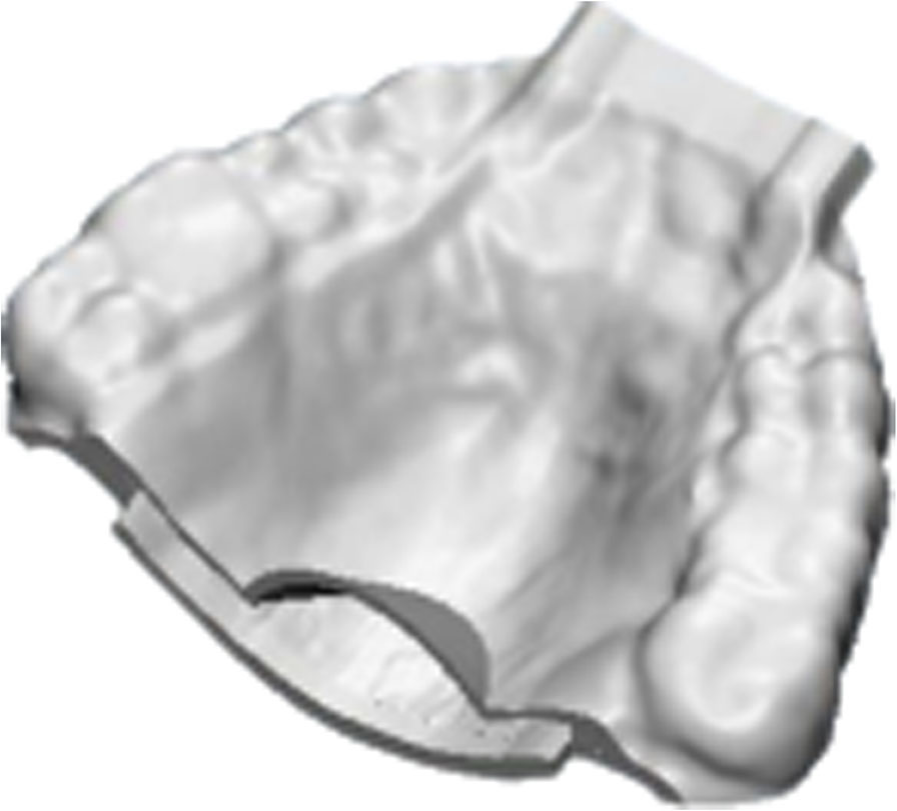
Prototype of customized maxillary oral appliance

#### Fabrication

Impressions of the upper and lower arch of the second group to be treated with the CMOA will be recorded. The cast obtained from the impression will be scanned for CAD designing with the help of CAD software. The design will be 3D printed in polymethylmethacrylate (PMMA ) material and will be delivered to the patient. All the patients will be advised to wear the appliance daily while sleeping at least for 6 h.

### Investigations

#### Polysomnography (PSG)

An overnight PSG will be performed in the Department of sleep medicine before and after one month and three months of intervention by MAD and CMOA. Recording of PSG will be scored as per the guidelines of the American Academy of Sleep Medicine. The event of apnea or hypopnea per hour will be calculated.

#### Oxygen saturation in the blood (SpO2)

Oxygen saturation in blood will be measured with a finger pulse oximeter at the time of PSG before and after one month and 3 months of intervention. Mean oxygen saturation, oxygen desaturation index (ODI), and proportion of time with SpO2 < 90%, will be evaluated.

#### Percentage of rapid eye movement (REM)

REM-AHI will be evaluated by calculating the number of events of AHI in REM sleep and dividing it by the amount of REM time with the help of PSG before and after one and three months of intervention

#### Electroencephalogram (EEG)

The pattern of EEG will be evaluated from PSG in the central lead C3-A2 at the apex of the cranium at 100 Hz sampling frequency to record Cortical arousals. Readings will be recorded before and after one month and three months of intervention for the participants of both study groups.

#### Electrocardiogram (ECG)

ECG patterns will be evaluated before and after one and three months of intervention. The ECG will be evaluated to identify anatomical, metabolic, ionic, and hemodynamic changes.

#### Oro-nasal airflow via a pressure transducer

A pressure transducer will be used to detect the mean respiratory disturbance index (RDI), before and after 1 and 3 months of intervention.

#### Epworth Sleepiness Scale (ESS)

ESS will be used to analyze the level of daytime sleepiness. Self-assessment will be done by the participants of both the study group before the intervention and after1 month and 3

months of intervention and score will be noted and compared

### Outcome measure

#### Primary outcome

The primary outcome will be the change in the score of AHI, SpO2, REM, EEG, ECG, RDI, and ESS from baseline to the end of 1 month of intervention by MAD and CMOA.

#### Secondary outcome

1. The change from baseline in the score of AHI, SpO2, REM, EEG, ECG, RDI, and ESS at the end of 3 months
2. The change in the score of AHI, SpO2, REM, EEG, ECG, RDI, and ESS at the end of 1 month and 3 months of intervention

### Safety evaluation

Adverse events (AEs), including AEs related to PMMA allergy, TMJ pain or muscle pain due to an increase in the vertical dimension of occlusion, gag reflex due to extension of appliances in the soft palate, and others will be recorded. For safety assessment, details of all such adverse events will be recorded by a research assistant in the case report form. Any serious adverse events will be reported within 24 h to the Institutional Ethics Committee.

### Data management/handling with missing data

Data entries undergo an automatic online check for plausibility and consistency. In case of implausibility, 'warnings' will be produced. A responsible investigator will be obliged either to correct the implausible data or to confirm its authenticity and to give an appropriate explanation. If not corrected, the data will be flagged, enabling a convenient check of all questionable entries. A responsible monitor will check all flagged data and generate questions that are sent back to the responsible investigator. The investigator will resolve all “discrepancies.”

Further checks for plausibility, consistency, and completeness of data will be performed after the completion of the study. Queries will be generated based on these checks, combined with a visual control by a responsible monitor/data manager.

All missing data or inconsistencies will be reported back to the sites and clarified by the responsible investigator. If no further corrections are to be made in the trial database it will be declared closed and used for statistical analysis.

For patients with incomplete follow-up, time to last follow-up date will be used as the censoring time in the analysis of time-to-event data. Missing data of continuous outcomes over time will be handled via the multi-level approach making the implicit assumption that data are missing at random, thus not requiring any direct imputation of missing continuous data. The robustness of this assumption will be explored in sensitivity analyses using pattern mixture models assuming that data are not missing at random. Otherwise, no imputation of missing data will be conducted.

In cases of withdrawal, the reason must be recorded and documented. For patients with incomplete follow-up, time to last follow-up date is used as the censoring time in the analysis of time-to-event data.

### Auditing the data

The investigators agree to allow the auditors/inspectors/monitors to have direct access to the trial records for review, being understood that this personnel is bound by professional secrecy, and as such will not disclose any personal identity or personal medical information. The investigator will make every effort to help with the performance of the audits and inspections, giving access to all necessary facilities, data, and documents.

### Statistical analysis

The baseline measures before intervention will be compared with the measures obtained after 1 month and 3 months of intervention. Descriptive and analytical statistics will be done. The data will be presented in mean and standard deviations. The normality of data will be tested by the Shapiro-Wilk test. If the data follows normal distribution parametric tests (Independent sample *t*-test and paired *t*-test) will be used and if the data does not follow normal distribution non-parametric tests (Mann-Whitney *U* test and Wilcoxon signed rank test) will be used. The statistical significance will be set at *p*< 0.05. SPSS (Statistical Package for Social Sciences) Version 20.1 (IBM Corporation, Chicago, USA) will be used as statistical software. The inverse probability censoring weighting (IPCW) approach will be done to estimate a treatment effect in the hypothetical scenario.

We expect to confirm the efficacy of the CMOA in managing moderate OSA against MAD by monitoring AHI, SpO2, REM, EEG, ECG, oro-nasal airflow via a pressure transducer, and ESS before and after 1 month and 3 months.

## Discussion

CPAP and OA are the standard treatment modalities for OSA. Zhang M et al. and Schwartz M et al. performed a meta-analysis on the effectiveness of oral appliances versus continuous positive airway pressure in treating OSA. They concluded that comparatively, CPAP is more efficient in reducing AHI, but it has significantly lower compliance resulting in no differences with MAD in quality of life, cognitive, or functional outcomes [19, 20].

Several designs of OA for managing moderate OSA have been documented, among which MAD is the most effective and recommended one [21, 22]. This study is an attempt to benchmark the CMOA as a treatment modality in managing cases with moderate OSA.

Cardinal features of the CMOA are-

- The overextension in the soft palate area for its support
- A bulge on its palatal surface to prevent the backfall of the tongue
- A hole in the anterior region to promote continuous inflow of air
- The hollowness of the plate to direct airflow towards the pharynx
- Use of CAD-CAM for precision

The design of this CMOA, recommends an increase in vertical dimension by 2 mm, which facilitates the forward and downward movement of the mandible that contributes to increasing the airflow by maintaining the patency of the airway. Patients of the age group 40-50 years often show the loss of vertical dimension of occlusion, this appliance will also help to regain the vertical dimension and re-establish the original centric relation in such patients [23]. Thus will simultaneously treat the symptoms of Temporomandibular disorders (TMD) if present due to loss of vertical dimension. This appliance will not lead to any changes in dentition as seen among patients treated with MAD.

Evaluating AHI by PSG is considered to be a gold standard for diagnosing and grading OSA. Events of Obstructive apnea (10 s cessation of airflow) and hypopnea (50% or less than 50% reduction in airflow) will be recorded as per the manual of the American Academy of sleep medicine [24]. Isacsson G et al in 2016 compare the effect of monobloc and bibloc appliances on AHI. AHI showed mean declined values of 12.7 and 13.8 for monobloc and bibloc, respectively [25].

However, Otero et al. stated that alone AHI is not sufficient to grade the severity of OSA [26]. And hence along with AHI, SPO2, REM, EEG, ECG, and oro-nasal airflow, ESS will also be evaluated in the present study to record the severity of OSA before and after one and three months of intervention by MAD and CMOA.

Fall by at least 4% in the mean oxygen saturation value is considered to be desaturation. The mean desaturation value in an hour during sleep is the Oxygen desaturation index (ODI). ODI is considered to have prognostic value as the complications and mortality of OSA are related to nocturnal hypoxia. Fietze et al. evaluated ODI for OSA patients and found a correlation between ODI and AHI among OSA patients [27]. Temirbekov D et al. in 2018 suggested that ODI is equally valuable as AHI in diagnosing and grading the OSA [28].

REM will be evaluated among subjects by calculating REM AHI. If REM AHI is greater than 5 events/hour, and the ratio of REM AHI/non-rapid eye movement (NREM) AHI is > 2, then the patient will be considered to have REM-OSA [29]. It has been proved, that in REM sleep autonomic nervous system and cardiorespiratory changes are more, which indicates that the cardiometabolic consequences are more worse in patients with high REM AHI [30]. According to Alzoubaidi M et al, the incidences of AHI are higher during REM sleep because of the decreased tone of genioglossus muscle secondary to the cholinergic mediated inhibition of the hypoglossal nerve [31]. Nishio Y et al. 2019 reported that OA contributed to reducing the apnea index (AI) during REM sleep but did not show a significant reduction in reducing the hypopnea index (HI) [32].

EG is an objective method of analyzing the changes in cortical activity. The present study will evaluate the efficacy of CMOA in normalizing EEG among study participants. Xiromeritis AG in 2011 found the slowing of EEG among OSA patients. He reported improvement in daytime sleepiness after CPAP therapy but found decreased alpha and increased delta relative power that indicated persistent brain dysfunction. He further added that hypoxia is a major cause of brain dysfunction [33]. Other studies have reported the slowing of EEG more prominently in REM sleep that makes sure that OSA during REM sleep leads to greater degrees of hypoxemia [34].

Patients with OSA show unusual ECG changes due to hemodynamic stress. Khalil et al. have mentioned a common feature of late depolarization of hypertrophied right outflow tract and/or left anterior fascicular block among patients with OSA [35]. The prevalence of this particular feature makes ECG a diagnostic tool in evaluating and grading the OSA. In the present study ECG will be monitored for any changes in its unusual pattern after intervention with the CMOA.

Alvarez D et al in 2009 found strong coordination between SPO2 and EEG finding in OSA patients [36]. This indicates that the values of the parameters that will be evaluated in the present study will be either direct proportion or inversely proportional to each other. That generates the secondary objective of the present study which will be to establish the relationship between these parameters for diagnosing and grading OSA.

RDI is the number of AHI per hour of total sleep time. A nasal transducer will be used to detect the variation in nasal pressure. Murray Johns developed ESS in 1991 to evaluate daytime sleepiness which has shown a strong correlation with OSA [37]. It is a numerical scale in which a score of > 10 suggests the presence of sleepiness. Many studies have used this scale to diagnose and analyze the treatment outcome [38–41]. In the present study, ESS will be evaluated before intervention and after one month and three months of intervention and will be compared to analyze the efficacy of customized maxillary oral appliances over MAD.

The singular tool should be avoided to stratifying any treatment modality and hence along with AHI, SPO2, REM, EEG, ECG, and oro-nasal airflow, ESS will also be evaluated to confirm the results before introducing this new design of maxillary customized oral appliances in the field of Sleep medicine for managing moderate OSA.

### Trial status

The current protocol version is 1, July 31st, 2020. Recruitment began on August 1, 2020, and is anticipated to be complete by June 1, 2021. As of November 2, 2020, Twenty participants have been included.

## Data Availability

The data will be shared after the trial is finished.

## Abbreviations

OA: Oral appliance
OSA: Obstructive sleep apnea
CPAP: Continuous positive airway pressure
RCT: Randomized controlled trial
AHI: Apnea/hypopnea index
MAD: Mandibular advancement device
CMOA: Customized maxillary oral appliance
TMD: Temporo-mandibular disorder
BMI: Basal metabolic index
RDI: Respiratory disturbance index
ESS: Epworth Sleepiness Scale
ECG: Electrocardiogram
EEG: Electroencephalogram
REM: Rapid eye movement
SpO_2_: Oxygen saturation in the blood
PSG: Polysomnography
PMMA: Polymethylmethacrylate
ODI: Oxygen desaturation index
3D: 3 Dimension
CAD-CAM: Computer-Aided Designing–Computer-Aided Manufacturing
NREM: Non-rapid eye movement
